# Effect of Resveratrol on Markers of Oxidative Stress and Sirtuin 1 in Elderly Adults with Type 2 Diabetes

**DOI:** 10.3390/ijms24087422

**Published:** 2023-04-18

**Authors:** Beatriz Isabel García-Martínez, Mirna Ruiz-Ramos, José Pedraza-Chaverri, Edelmiro Santiago-Osorio, Víctor Manuel Mendoza-Núñez

**Affiliations:** 1 Posgrado en Ciencias Biológicas, Universidad Nacional Autónoma de México (UNAM), Unidad de Posgrado, Mexico City 04510, Mexico; isabelgm06@gmail.com; 2 Research Unit on Gerontology, FES Zaragoza, Universidad Nacional Autónoma de México (UNAM), Mexico City 09230, Mexico; 3 Department of Biology, Faculty of Chemistry, Universidad Nacional Autónoma de México (UNAM), Mexico City 04510, Mexico; pedraza@unam.mx; 4 Hematopoiesis and Leukemia Laboratory, Research Unit on Cell Differentiation and Cancer, FES Zaragoza, Universidad Nacional Autónoma de Mexico (UNAM), Mexico City 09230, Mexico

**Keywords:** oxidative stress, sirtuin 1, resveratrol, type 2 diabetes

## Abstract

Type 2 diabetes (T2D) affects a large part of the adult population and impairs its quality of life. Because of this, natural compounds with antioxidant, anti-inflammatory and hypoglycemic properties have been used as adjuvants. Among these compounds, resveratrol (RV) stands out, a polyphenol that has been studied in several clinical trials, the results of which are controversial. We conducted a randomized clinical trial on 97 older adults with T2D to evaluate the effect of RV on oxidative stress markers and sirtuin 1, using doses of 1000 mg/day (EG1000, *n* = 37) and 500 mg/day (EG500, *n* = 32) compared with a placebo (PG, *n* = 28). Biochemical markers, oxidative stress and sirtuin 1 levels were measured at baseline and after six months. We observed a statistically significant increase (*p* < 0.05) in total antioxidant capacity, antioxidant gap, the percentage of subjects without oxidant stress and sirtuin 1 levels in EG1000. In the PG, we observed a significant increase (*p* < 0.05) in lipoperoxides, isoprostanes and C-reactive protein levels. An increase in the oxidative stress score and in the percentage of subjects with mild and moderate oxidative stress was observed too. Our findings suggest that 1000 mg/day of RV exerts a more efficient antioxidant effect than 500 mg/day.

## 1. Introduction

Type 2 diabetes (T2D) is a metabolic and chronic disease that arises due to inefficiency in the body in the use and/or production of insulin, whose hormone is involved in glucose metabolism, which triggers hyperglycemia [1]. T2D is a public health problem in the world. In this sense, a prevalence of 10.5% (536.6 million people) has been estimated, projecting an increase to 12.2% (783.2 million) by 2045 in the population aged 20 to 79, being significantly higher in older adults [2]. This is worrisome since, in the long term, hyperglycemia gives rise to the appearance of micro- and macrovascular complications that affect the functionality of organs such as the eyes, kidneys and heart, which has a negative impact on the quality of life of those with T2D in addition to representing a huge burden on the health system [3]. It has been shown that the hyperglycemia that characterizes T2D is partially responsible for the excessive production of free radicals (FR) and/or reactive oxygen species (ROS), which cause oxidative damage to biomolecules since excess glucose in the blood auto-oxidizes and generates ROS; therefore, if they are not counteracted by the antioxidant systems, they alter the physiology of the cells, whose biochemical disorder is defined as oxidative stress (OS) [4]. Excess ROS interferes with nerve conduction and alters the functionality of renal cells, favoring the appearance of neuropathy and nephropathy, which are highly recurrent complications in T2D [5]. In addition, the elevated production and accumulation of ROS also occur during the aging process, which is why OS levels are significantly higher in older adults with T2D [6,7].

In this context, alternative therapeutic options have been proposed to attenuate oxidative damage and thereby delay the onset of T2D complications. In this sense, one of the most widely used is resveratrol (RV), a compound from the stilbenes family that, due to its polyphenolic nature, is capable of donating electrons to FR and ROS, which is why it is considered a powerful antioxidant [8,9,10]. In this regard, several investigations have shown that RV could be useful in the treatment of chronic non-communicable diseases, whose pathophysiology involves OS and inflammation, as occurs with neurodegenerative, cardiovascular and metabolic diseases since it also has anti-inflammatory properties [11,12,13,14,15]. The therapeutic mechanisms of RV are largely attributed to its ability to activate sirtuin 1 (SIRT1), a deacetylase enzyme that, once activated, stimulates AMP-dependent protein kinase (AMPK) and is the interaction between both proteins, which manages to improve biogenesis and mitochondrial function, increase insulin sensitivity, attenuate oxidative damage and regulate metabolic homeostasis [16,17,18]. In some studies, carried out in animal models with T2D, it has been shown that RV exerts antioxidant, anti-inflammatory and even hypoglycemic effects; however, clinical trials have shown controversial results [19,20,21]. Such discrepancies have been attributed to differences in the age and health conditions of the participants, the duration of the interventions and the doses used, which range from 10 mg/day to 3000 mg/day [22]. In this sense, in a systematic review and meta-analysis carried out by our research group, we found that doses greater than 100 mg/day have a hypoglycemic effect, although this effect is greater with doses of 500–1000 mg/day [23]. This suggests that the therapeutic properties of RV occur in a dose-dependent manner, so the aim of this study was to evaluate the effect of the oral administration of RV at a dose of 500 mg/day compared with 1000 mg/day on markers of SIRT1 and SIRT1 in older adults with T2D.

## 2. Results

**Figure 1:**
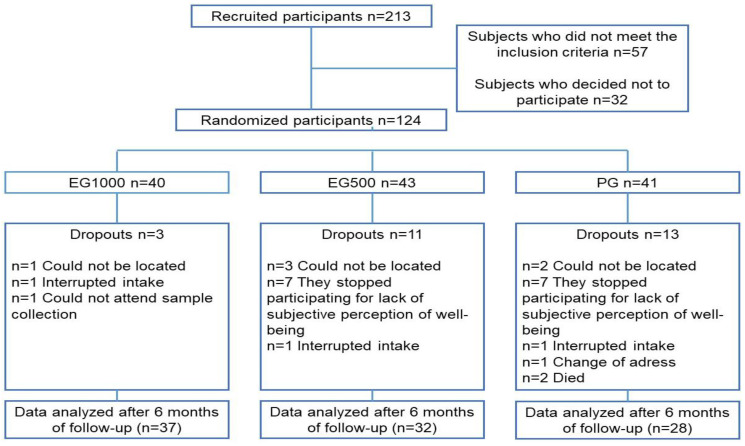
General study flowchart. Abbreviations: EG1000, group supplemented with 1000 mg/day of RV; EG500, group supplemented with 500 mg/day of RV; PG, placebo group.

Figure 1 presents the general scheme of the study.

### 2.1. Clinical Parameters (BMI and Blood Pressure)

Table 1 presents the data on the clinical and anthropometric characteristics of the three study groups. No differences were observed between the groups at the end of the 6-month follow-up.

### 2.2. Biochemical Parameters

Regarding biochemical parameters, a statistically significant decrease (*p* < 0.05) was observed in triglyceride levels in the EG1000 after the intervention (baseline, 170 ± 69 vs. six months, 147 ± 46 mg/dL). Likewise, a significant increase (*p* < 0.05) was found in the CRP values in the PG after 6 months of follow-up (baseline, 0.37 ± 0.4 vs. six months, 0.48 ± 0.5 mg/dL); as can be seen in Table 2, no change was observed in the rest of the biochemical parameters evaluated.

### 2.3. OS Markers

A statistically significant increase (*p* < 0.05) was found in the concentration of lipoperoxides and 8-isoprostanes in the PG after the intervention (LPO: baseline, 0.219 ± 0.07 vs. six months, 0.282 ± 0.07 µmol/L; 8-Iso: baseline, 61 ± 24 vs. six months, 76 ± 35 pg/mL). We also observed a significant increase (*p* < 0.05) in TAC and GAP in EG1000 after RV treatment (TAC: baseline, 1003 ± 247 vs. six months, 1225 ± 249 µmol/L; GAP: baseline 283 ± 242 vs. six months, 434 ± 234). Likewise, SOD activity decreased in the PG (baseline, 171 ± 15 vs. six months, 167 ± 10 IU/L; *p* < 0.05). A statistically significant increase in the oxidative stress score (OSS) was also found (baseline, 1.9 ± 1 vs. six months, 2.8 ± 1; *p* < 0.05). The rest of the evaluated markers did not show significant changes after six months (Table 3).

Regarding the OS index (OSI), an increase was found in the percentage of individuals without OS in EG1000 (baseline, 13 vs. six months, 35%; *p* < 0.05). Likewise, the percentages of moderate OS (MOS) and severe OS (SOS) decreased (MOS: baseline, 27 vs. six months, 13%; SOS: baseline, 22 vs. six months, 83%; *p* < 0.05) after the intervention. In the EG500, a significant increase (*p* < 0.05) was found in the percentage of individuals without OS (baseline, 10 vs. six months, 28%). In the PG, we observed a significant decrease in the percentage of subjects without OS (baseline, 21 vs. six months, 7%; *p* < 0.05) and in subjects with mild OS (baseline, 43 vs. six months, 29%; *p* < 0.05), while the percentage of individuals with moderate OS increased after the intervention (baseline, 29 vs. six months, 54%; *p* < 0.05), as seen in Table 4.

### 2.4. SIRT1 Concentration

**Figure 2:**
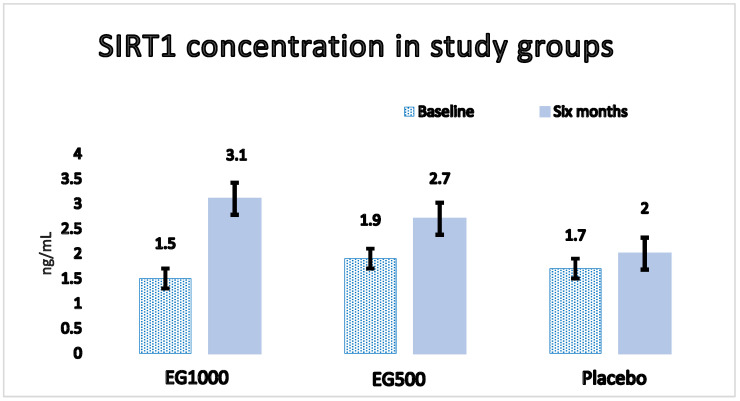
SIRT1 concentration in study groups: baseline and after six months. Data are presented as mean ± SE. A statistically significant increase (1.5 ± 0.15 vs. 3.1 ± 0.20 ng/mL; *p* < 0.05) in EG1000 is observed after follow-up.

Regarding the concentration of SIRT1, we observed a statistically significant increase in EG1000 (baseline, 1.5 ± 1 vs. six months, 3.1 ± 2 ng/mL; *p* < 0.05) after six months of follow-up, as shown in Figure 2.

### 2.5. Adverse Events

None of the participants reported adverse events attributable to RV administration.

## 3. Discussion

In recent decades, RV has been one of the most studied nutraceuticals in numerous research groups. In this sense, in some experiments carried out in vitro and in animal models, its antioxidant, anti-inflammatory, hypoglycemic, neuroprotective, anti-aging and even antineoplastic effects have been demonstrated, for which reason it is recognized as a product of natural origin with great potential as a therapeutic agent for cardiovascular, neurodegenerative and metabolic diseases [24,25], among which T2D stands out, whose prevalence, incidence and lethality have family, social and economic implications. T2D represents a great burden on health systems because of its high frequency of complications, which increases the need for hospital care and severely affects the resources of diabetic people and their families, in addition to deteriorating their quality of life [26]. Despite the efforts of the medical community with conventional treatments, it has not been possible to reduce the prevalence and incidence of T2D or the development of micro- and macrovascular complications in diabetic subjects, which is why complementary antioxidant and anti-inflammatory treatments have been proposed [27]. In this regard, although these effects of RV have been demonstrated in animal models with induced diabetes, the evidence in clinical trials shows inconsistent results, so research on the potential beneficial effects of RV on humans is a relevant and current topic [19,20,21].

On the other hand, it has also been proposed that RV could exert a hypotensive effect due to its ability to induce the production of nitric oxide (NO), a potent vasodilator that is produced in the vascular endothelium [28,29]. However, this effect has only been observed in hypertensive people. In accordance with the above, in our clinical trial, we did not observe such an effect, which suggests that RV could have a specific modulatory effect that responds to high blood pressure figures. It has also been reported that RV stimulates the coactivator of the receptor gamma-1α, which is activated by peroxisomal proliferators (PGC-1α), a transcription factor that regulates genes that modulate the storage of body fat; thus, through this pathway, it could induce weight loss and decrease BMI [30]. This effect has been observed in studies carried out on subjects with obesity (BMI > 30 kg/m^2^) [30,31]; however, in our study, a decrease in BMI was not observed because our population did not present with obesity. Therefore, this result also suggests a specific modulating effect of RV on lipolysis, similar to that observed on blood pressure.

Regarding the effect of RV on the control of T2D, RV has been shown to reduce blood glucose levels due to the increase in the expression of the glucose transporter (GLUT4) in skeletal muscle cells, improving its uptake, use and storage [15]. It has also been proposed that RV protects the islet cells of Langerhans from oxidative damage, increases their viability, restores β cell secretory functions and normalizes insulin secretion, which gradually restores the signaling pathway of insulin and contributes to glycemic control [12,32]. In this sense, in some clinical trials, it has been reported that, with doses between 200 and 1000 mg/day of RV, the levels of glucose, insulin, HbA1c and insulin resistance are reduced [33,34,35,36] in subjects with T2D younger than 50 years. Likewise, in two randomized double-blind placebo-controlled clinical trials carried out on individuals older than 60 years, using doses of 500 and 800 mg/day, respectively, a hypoglycemic effect was demonstrated after 4 and 8 months of treatment [37,38]. This effect in older adults has been consistent, as reported in the meta-analyses published by Hausenblas et al. (2014) [39] and Liu et al. (2014) [40]. In contrast, in our study, we did not observe significant changes in glucose and HbA1c levels after 6 months of treatment, results that coincide with what was reported in two double-blind randomized clinical trials, in which 150 mg/day doses of RV were administered for 30 days in subjects with DM2 younger than 50 years [41,42]. These results may be due to the dose and duration of the interventions, which may be insufficient to observe the hypoglycemic effects of RV. The results of our clinical trial also coincide with those obtained in two double-blind randomized clinical trials in which doses of 500 and 1000 mg/day of RV were administered for 6 months and 5 weeks, respectively, both in subjects older than 60 years [43,44], in whom no significant changes were found in insulin sensitivity, glycemic control, glucose levels or HbA1c. Likewise, in the meta-analysis conducted by Jeyaraman et al. (2020), they also did not find a hypoglycemic effect in subjects 60 years of age or older [45]. The findings of the mentioned studies agree with what was observed in a systematic review and meta-analysis carried out by our research group, in which it was found that the effects of RV are notably less on people over 60 years of age compared with younger people since, in parallel with the low bioavailability of RV, adults over 60 years of age present with absorption problems at the intestinal level and have a reduced capacity to metabolize RV, in addition to OS related to aging, for which reason it has been suggested that doses at high rates and interventions of longer duration (6 months or more) be used in this age group [46].

Regarding the lipid-lowering effect, we observed a statistically significant decrease in the serum concentration of triglycerides in EG1000, which can be attributed to the ability of RV to decrease the absorption of fatty acids and de novo lipogenesis, in addition to increasing fat mobilization and inducing the β-oxidation of fatty acids [47]. This lipid-lowering effect is consistent with what was observed in the meta-analysis carried out by Cao et al. (2022), who found that the oral administration of RV significantly reduces serum triglyceride levels [48].

In the present study, a significant increase in CRP was also observed in the PG after follow-up, which may be due to chronic low-grade inflammation arising as a consequence of the hyperglycemia that characterizes T2D [49]. In this sense, it has been shown that hyperglycemia promotes the excessive formation of advanced glycation end products (AGEs), and these activate nuclear factor κB (NFκB), which triggers the transcription of pro-inflammatory cytokine promoter genes, and, in turn, the latter stimulate the production of CRP in the liver, which is why the protein is elevated in individuals with T2D [49,50], as was observed in the PG. In contrast, in EG1000, no significant changes were observed after RV administration, as it is able to block NFκB activation and prevent increased CRP production [51].

Regarding OS markers, RV has been shown to exert antioxidant effects through two mechanisms: (i) through direct interaction with RL and ROS and (ii) through its ability to increase the activity of antioxidant enzymes [52]. In this sense, the first mechanism is due to the polyphenolic nature of RV, mainly the free hydroxyl group in position 4, since the extraction of its hydrogen allows electrons to be mobilized throughout the chemical structure of RV and gives it redox characteristics, which allow it to interact with FR and ROS, making it an excellent scavenger of hydroxyl, peroxide and superoxide [52,53]; as a consequence of this activity, it can minimize or prevent the oxidation of polyunsaturated fatty acids that are abundant in cell membranes, protecting them from oxidative damage [52,53,54]. In line with this, the results of two randomized double-blind clinical trials found a significant decrease in malondialdehyde (MDA) levels, a direct biomarker of lipid peroxidation in membranes, with doses of 200 and 500 mg/day of RV in subjects with T2D [33,55]. In our clinical trial, we observed a statistically significant decrease in LPO (MDA) levels in EG1000 and an increase in the same marker in the PG, which is consistent with the antioxidant effect of RV reported in other studies. Likewise, we observed a significant increase in 8-isoprostane (8-Iso) concentration in the PG and a significant decrease in EG1000, demonstrating the antioxidant effect of RV; 8-Iso is formed as a secondary product of lipid peroxidation, whose molecule is similar in structure to prostaglandins and is currently considered the gold standard for measuring oxidative damage to membranes [56], which agrees with the results observed in the PG. The second mechanism of RV is through the increase in the activity of antioxidant enzymes (SOD, GPx, Cat), responsible for eliminating excess FR and ROS to prevent oxidative damage [57]. These three enzymes work together to protect cells from ROS. First, SOD dismutates the superoxide anion into hydrogen peroxide, and later, GPx and Cat degrade it into oxygen and water [58]. This effect is achieved through its interaction with erythroid-derived nuclear factor 2 (Nrf2), a transcription factor that modulates the expression of genes containing antioxidant response elements (AREs). The activation of the Nrf2/ARE pathway promotes the expression of antioxidant genes and stimulates the production of SOD, GPx and Cat enzymes, whose function is to eliminate ROS [58,59]. In this sense, it has been observed that RV activates Nrf2 through the activation of AMPK, which, in turn, stimulates the Nrf2/ARE pathway and, as a result, increases the production of antioxidant enzymes [58,59,60]. In our study, we found a non-significant increase in SOD activity in EG1000 and a statistically significant decrease in the PG, which agrees with what was stated above, as well as with the results of two randomized double-blind clinical trials in which an increase in SOD activity was observed with RV treatments in doses of 500 and 800 mg/day, and a decrease in the activity of the enzyme was observed in groups treated with a placebo [55,61]. The authors of these studies also reported that TAC was elevated after RV administration, as occurred in the EG1000 of our clinical trial. In addition to this, we observed that, after the follow-up, the antioxidant gap (GAP) increased in the EG1000. The GAP is a calculation that provides information about the antioxidant activity of plasma components other than uric acid and albumin, such as α-tocopherol, ascorbic acid, transferrin and other antioxidants obtained from the diet, for example, RV [62], which explains the increase in GAP in the EG1000 after six months of orally administering 1000 mg/day of RV.

Regarding the oxidative stress score (OSS), a statistically significant increase was observed in the PG, and a decrease was observed in EG1000. In this sense, the decrease in the OSS is the result of an increase in TAC and GAP, which counteracts ROS and attenuates oxidative damage to the membranes, reflected in the decrease in LPO and 8-isoprostane levels. Although the activity of the SOD and GPx enzymes did not increase significantly with the OSS value, we can infer that the antioxidant enzymes evaluated act in an integral and efficient manner to counteract high ROS concentrations. Regarding the oxidative stress index (OSI), a decrease was found in the percentage of individuals with moderate and severe OS in EG1000, while in the PG, the proportions of subjects without OS and mild OS decreased, and that of moderate OS increased. This is consistent with what has been reported in the literature since the group that received 1000 mg/day of RV showed a higher antioxidant effect compared with the subjects that received 500 mg/day treatment.

We also observed a statistically significant increase in SIRT1 concentrations in the EG1000, which is consistent with the proposed mechanism of RV [63,64]. In this sense, SIRT1 has an effect on the regulation of autophagy, mitophagy, mitochondrial biogenesis, the expression of antioxidant enzymes and the suppression of NFκB, among others [63]. Likewise, the production, expression and activity of SIRT1 can be modified by RV in an allosteric way [64] so that an increase in SIRT1 levels provides indirect information on the effects of RV [63]. On the other hand, when SIRT1 is stimulated, it deacetylates and activates the LKB1 protein kinase, which phosphorylates and activates AMPK, thereby attenuating oxidative damage, improving insulin resistance and restoring energy homeostasis [64]. Regarding the role of SIRT1 in energy metabolism, scientific evidence suggests that it increases oxidative phosphorylation through the deacetylation of PGC-1α and thereby functions as a regulator of mitochondrial biogenesis in the liver and muscle [65]; PGC-1α controls the expression of genes that regulate biogenesis and mitochondrial activity, decreasing lipid accumulation and increasing glucose uptake, thereby improving energy metabolism [66]. In relation to the antioxidant effect of RV through SIRT1, it has been shown that SIRT1 stimulates AMPK, and this negatively regulates NADPH oxidase, the enzyme responsible for producing ROS, and induces an increase in the expression of SOD, the final result of which is a decreased OS [67]. SIRT1 also regulates the acetylation of FOXO family transcription factors, involved not only in lipid and glucose metabolism but also in the response to OS. In this regard, it has been observed that the SIRT1–AMPK interaction stimulates the transcriptional activity of FOXO3, and the expression of manganese-dependent SOD (MnSOD) is induced in cells that overexpress FOXO3 [68]. Likewise, the transcriptional activity of FOXO3 triggers the inhibition of NADPH oxidase, and, therefore, the production of ROS is reduced; therefore, through SIRT1 and FOXO3, the RV decreases the amount of ROS and increases the expression of MnSOD, mechanisms through which it attenuates OS [69]. In addition, it has been proposed that the complex formed by the SIRT1-FOXO3-PGC-1α interaction activates Nfr2; as has been pointed out, it is a transcriptional regulator of the expression of genes involved in the antioxidant response since it promotes MnSOD production and other enzymes that provide antioxidant protection to mitochondria [70]. The mentioned mechanisms explain how SIRT1 mitigates OS and prevents oxidative damage to cells, which has been evidenced in cell cultures, where it has been observed that a moderate overexpression of SIRT1 protects cells from OS, while a deficiency in SIRT1 induces an increase in the production of ROS [69,70,71]. However, the relationship between SIRT1 and OS is more complex than it seems since SIRT1 is involved in redox-dependent cellular processes such that, just as SIRT1 can influence the redox state of cells, the redox state is capable of altering the production and enzymatic activity of SIRT1 through post-translational modifications (phosphorylations, S-nitrosylations and carbonylations). In some cases, these alterations can be detrimental to cells and influence the pathogenesis of chronic diseases involving SG; such is the case for T2D [71]. In this sense, the activation of SIRT1 by RV induces the proposed antioxidant mechanisms only if adequate redox conditions are achieved since SIRT1 is a protein sensitive to the redox state, and if this does not foster the adequate conditions, activation by RV is insufficient in exerting antioxidant effects. Considering the above, the results of our investigation demonstrate a more efficient antioxidant effect of RV at doses of 1000 mg/day compared with doses of 500 mg/day, which also coincides with an increase in SIRT1 levels induced by RV.

There are several foods and beverages that contain RV: (i) 150 mL of red wine contains up to 2.15 mg; (ii) 250 mL of red grape juice contains 1.25 mg; (iii) 250 g of dehydrated red grapes contains 1.6 mg; (iv) 150 g of frozen strawberries contains 1.56 mg; (v) 125 g of blueberries contains 2.41 mg; and (vi) 250 g of peanuts contains 1.3 mg [10,72]. The daily consumption of these foods can provide <10 mg of RV, which is insufficient to exert therapeutic effects [72]. In this regard, our study did not control the amount of RV consumed through the diet; however, the RV obtained from food does not reach sufficient amounts to influence the results obtained. In addition, considering that the participants are diabetic, the consumption of said foods would have to be assessed since most of them contain sugar, and their consumption can trigger the elevation of blood glucose levels.

Finally, none of the participants reported adverse events during the intervention. In this sense, RV is considered a safe compound for human consumption since it does not exert secondary or toxic effects at relatively high doses (1000–2000 mg/day). In fact, with doses of 2.5 to 5 g/day, only mild gastrointestinal effects occur, such as nausea, vomiting and diarrhea [54,73].

## 4. Materials and Methods

A double-blind randomized clinical trial was carried out, previously approved by the Bioethics and Biosafety Committee of the Universidad Nacional Autónoma de México (UNAM) FES Zaragoza (FESZ/DEPI/CI/037/20) and registered in ISRCTN (ISRCTN15172592).

### 4.1. Population and Study Design

A call was made through social networks to recruit the study population according to the following selection criteria: men and women between 60 and 74 years of age, with a clinical diagnosis of T2D treated with metformin and/or glibenclamide as a hypoglycemic agent, without kidney or liver damage, residents of Mexico City. A total of 213 candidates agreed to participate in the study, of which 124 met the established inclusion criteria and signed the informed consent (Figure 1). Weight, height, body mass index (BMI) and blood pressure were measured for all participants entering the study. Blood samples were also taken to evaluate biochemical parameters, such as glucose, HbA1c, total cholesterol, HDL cholesterol, triglycerides, uric acid, urea and complete blood count, as well as for the measurement of OS markers (lipoperoxidation; 8-isoprostanes; activities of the antioxidant enzymes superoxide dismutase, glutathione peroxidase and catalase; and total antioxidant capacity), SIRT1 levels and C-reactive protein (CRP).

After performing the baseline measurements of all the clinical and biochemical parameters, a person outside our research team randomly assigned the 124 participants using the online tool “randomizer.com”, integrating the following 3 study groups: (i) experimental group 1000 mg/day (EG1000), *n* = 40 participants who received 1000 mg/day of trans-resveratrol, divided into 2 capsules, each containing 500 mg; (ii) experimental group 500 mg/day (EG500), *n* = 43 participants who were administered 500 mg/day of trans-resveratrol, divided into 2 capsules, each containing 250 mg; (iii) placebo group (PG), *n* = 41 participants who received daily 2 capsules identical in appearance to those of the other two groups but whose content was a placebo (Figure 1). Treatments were performed with synthetic trans-resveratrol (>99% purity) and a mixture of crystalline microcellulose with magnesium stearate as anti-caking, dispersing and stabilizing agents. The aforementioned mixture of excipients was used as a placebo since both compounds are considered “safe for human consumption” by the FDA, as well as inert. The treatments for the three study groups were prepared by “Productos Naturales Anáhuac S.A. de C.V.” Randomization and assignment to groups were blinded for the participants and for the personnel in charge of the investigation until the final analysis of all the data collected during the study was carried out. The treatments (capsules) were delivered to the participants in an identical presentation in shape, color, size and weight, regardless of the group to which they were administered. In addition, they were contained in opaque bottles labeled with the names of the participants, which were delivered monthly by a person unrelated to the investigation. The deliveries were made out for 6 months, and adherence to the treatment was verified by counting the remaining capsules in the bottles. During the intervention, the research team maintained telephone contact with the participants to identify possible unwanted effects. After 6 months, all the initial measurements were performed again; however, during the follow-up, 27 participants dropped out of the study for various reasons, so only 97 data were analyzed (EG1000 *n*= 37, EG500 *n*= 32, PG *n* = 28), as presented in Figure 1.

### 4.2. Measurement of Clinical Parameters (Weight, Height, BMI and Blood Pressure)

The weight of the participants in underwear was measured using a calibrated scale (Torino, Tecno Logica Mexicana, Mexico City, Mexico^®^). To measure the height, the participants were placed standing with their heels together, keeping the buttocks, shoulders and head in contact with an aluminum stadiometer graduated in millimeters (SECA^®^, Hamburg, Germany), with their eyes facing forward and the Frankfurt plane parallel down. The BMI was calculated by dividing the weight (expressed in kg) by the square of the height (expressed in meters) (IMC = kg/m^2^). Blood pressure was determined in accordance with the provisions of NOM-030-SSA-1999 for the prevention, detection, diagnosis, treatment and control of arterial hypertension. Each participant was asked to sit in a chair with a backrest, with one arm uncovered and flexed on a support. A stethoscope and a sphygmomanometer coupled with a mercury column were used to measure systolic and diastolic blood pressure [74].

### 4.3. Measurement of Biochemical Parameters

After fasting for 8 to 10 h, blood samples were obtained with vacutainer equipment in tubes (Beckton-Dickinson, NJ, USA) with an EDTA anticoagulant to perform a complete blood count and quantify the percentage of HbA1c. Likewise, samples were taken without an anticoagulant for biochemical tests (glucose, total cholesterol, HDL-cholesterol, triglycerides, uric acid, urea). Blood count was performed with a whole blood sample in a hematology analyzer (Spincell, Spinreact, Girona, Spain). The measurement of the biochemical parameters and the CRP was carried out with serum samples using an autoanalyzer (Selectra Junior, Siemens, Munich, Germany) through colorimetric and turbidimetry techniques, respectively. HbA1c was measured in EDTA anticoagulated blood samples via turbidimetry in the Selectra Junior autoanalyzer.

### 4.4. Measurement of OS Markers

Blood was drawn into heparinized tubes for the measurement of OS markers.

#### 4.4.1. Lipoperoxidation (LPO)

This determination was performed on a heparinized plasma sample, as described by Jentzsch [75]. We used malondialdehyde (MDA) as a lipid peroxidation marker since it is one of the main oxidation bioproducts of polyunsaturated fatty acids, as well as the main reactive molecule of thiobarbituric acid (TBA). Each MDA molecule contained in the sample reacts with two TBA molecules to form a pink-colored compound, whose absorbance is measured at 535 nm. To obtain the MDA concentration, the absorbance was interpolated in a calibration curve.

#### 4.4.2. 8-Isoprostane (8-Iso)

To measure the concentration of 8-isoprostane, we used plasma samples obtained from blood with EDTA. We also used the 8-isoprostane EIA kit (Cayman Chemical, Ann Arbor, MI, USA). The basis of this measurement is the competition between 8-isoprostane and an 8-isoprostane–acetylcholinesterase conjugate for the binding of a certain number of 8-isoprostane-specific antibodies. The amount of 8-isoprostane tracer remains constant, but the levels of 8-isoprostane in the sample vary such that the amount of tracer that binds to the specific antibody of the 8-isoprostane is inversely proportional to the concentration of 8-isoprostane in the sample. The antibody–8-isoprostane complex binds to a second antibody, and upon the addition of the corresponding substrate, a yellow-colored compound is formed, which is measured spectrophotometrically at 412 nm. The intensity of the color is inversely proportional to the amount of 8-isoprostane present in the analyzed sample.

#### 4.4.3. Superoxide Dismutase (SOD)

To measure SOD activity, a heparinized blood sample and the Ransod commercial kit (Randox Laboratories Ltd., Crumlin, UK) were used. The method is based on what was described by McCord and Fridovich [76], in which the reaction between xanthine and xanthine oxidase (XOD) produces superoxide radicals that react with 2-(4-iodophenyl)-3-(4-nitrophenol)-5-phenyltetrazolium to form formazan red. The SOD present in the sample neutralizes the superoxide radicals and inhibits the formation of formazan red, so the activity of the enzyme is directly proportional to the degree of inhibition. The kinetics of the reaction was measured spectrophotometrically at 505 nm.

#### 4.4.4. Glutathione Peroxidase (GPx)

To measure the GPx, the method reported by Plagia and Valentine [77] was used. A heparinized blood sample and the commercial Ransel kit (Randox Laboratories Ltd., Crumlin, UK) were required. The GPx present in the sample to be analyzed catalyzes the oxidation of glutathione (GSH) via cumene hydroperoxide in the presence of glutathione reductase (GR) and NADPH; then, the oxidized glutathione (GSSG) is converted into its reduced form with the concomitant oxidation of NADPH into NADP+. This reaction causes a decrease in absorbance, which was measured at 340 nm in a spectrophotometer.

#### 4.4.5. Catalase (Cat)

To quantify the activity of this enzyme, the method described by Aebi [78] was used, in which H_2_O_2_ is used as a substrate and the continuous decrease in its concentration is monitored using its decomposition due to the action of the enzyme. The decrease in absorbance at 240 nm was measured since said decrease is proportional to the catalase activity.

#### 4.4.6. SOD/GPx Ratio

The calculation was made using the values obtained for each enzyme. It is a theoretical model that provides information about the biochemical interactions of enzymes.

#### 4.4.7. Total Antioxidant Capacity (TAC)

In this technique, a sample of heparinized plasma and a commercial total antioxidant status kit (Randox Laboratories Ltd., Crumlin, UK) were used. For the measurement, the peroxidase enzyme is combined with H_2_O_2_ and 2,2′-azido-diethylbenzothialinsulfonate; this results in the production of the ABTS+ cation, which presents a bluish-green coloration. The antioxidants contained in the sample suppress this coloration, this being proportional to the concentration of antioxidants [79]. The kinetics of the reaction was measured at 600 nm. All spectrophotometric measurements were performed on a Multiskan Go Microplate spectrophotometer (Thermo Scientific, Denver, CO, USA).

#### 4.4.8. GAP Calculation

We applied the following formula to calculate the antioxidant gap (GAP):

GAP = (TAC − [(albumin (mmol) × 0.69) + uric acid (mmol)])

#### 4.4.9. Calculation of Oxidative Stress Score (OSS)

In this calculation, we used cutoff points previously established by our research group for the following markers: LPO ≥ 0.340 mmol/L, SOD ≤ 170 IU/mL, GPx ≤ 5500 IU/L, Cat ≤ 0.9 mmol/L, SOD/GPx ≥ 0.023 and GAP ≤ 190 mmol/L. For each datum outside the cutoff values, one point was awarded, and a sum was made to obtain the OSS. The OSS value is directly proportional to OS levels [62].

#### 4.4.10. OS Index (OSI)

This index was obtained by categorizing the OSS as follows [62]:

0 points: Without OS;

1–2 points: Mild OS;

3–4 points: Moderate OS;

5–6 points: Severe OS.

### 4.5. Measurement of SIRT1

SIRT1 quantification was performed using the double sandwich ELISA technique. We used a monoclonal anti-human SIRT1 antibody and a biotinylated detection antibody. Samples and biotinylated antibodies were added to ELISA plate wells and then washed with buffer. Then, avidin–peroxidase conjugate was added to the wells, and the corresponding substrate was added. The reaction mixture turned blue and then turned yellow upon the addition of acid. The absorbance of the colored compound was read at 450 nm on a Multiskan Go Microplate spectrophotometer (Thermo Scientific, Denver, CO, USA).

### 4.6. Statistical Analysis

Frequencies and percentages for qualitative variables, mean and standard deviation for quantitative variables were calculated. Comparisons were made using the repeated measures of ANOVA and McNemar, with a 95% confidence interval. A value of *p* < 0.05 was considered an indication of a statistically significant test. All statistical analyses were performed using the SPSS 21 software.

## 5. Conclusions

Our findings suggest that the consumption of RV at a dose of 1000 mg/day exerts a more efficient antioxidant effect than a dose of 500 mg/day, which coincides with a statistically significant increase in SIRT1 levels in older adults with T2D. This provides evidence suggesting that the consumption of RV in doses of at least 1000 mg/day for 6 months or more could be an adjunctive treatment that reduces the incidence of micro- and macrovascular complications linked to T2D.

## Figures and Tables

**Table 1:** Clinical parameters (BMI and blood pressure) at baseline and after six months in the study groups.

Parameter	EG1000 (*n* = 37)		EG500 (*n* = 32)		Placebo (*n* = 28)	
	Baseline	Six months	Baseline	Six months	Baseline	Six months
Age (years)	66 ± 6		63 ± 7		64 ± 5	
BMI (kg/m^2^)	27.6 ± 4.4	27.7 ± 4.2	27.8 ± 3.6	27.9 ± 4.0	28.3 ± 3.4	27.9 ± 3.0
SBP (mmHg)	127 ± 16	124 ± 12	128 ± 17	127 ± 14	127 ± 10	123 ± 10
DBP (mmHg)	84 ± 6	82 ± 9	84 ± 13	86 ± 8	82 ± 7	82 ± 9

BMI: body mass index, SBP: systolic blood pressure, DBP: diastolic blood pressure. Data are presented as mean ± SD. ANOVA of repeated measures.

**Table 2:** Biochemical parameters of the study groups at baseline and after six months.

Parameters	EG1000 (*n* = 37)		EG500 (*n* = 32)		Placebo (*n* = 28)	
	Baseline	Six months	Baseline	Six months	Baseline	Six months
Hemoglobin (g/dL)	14 ± 1	14 ± 2	14 ± 1	15 ± 2	14 ± 2	14 ± 2
Hematocrit (%)	45 ± 4	45 ± 4	45 ± 6	46 ± 5	45 ± 6	46 ± 5
Glucose (mg/dL)	169 ± 73	186 ± 83	185 ± 65	203 ± 77	189 ± 74	184 ± 67
Urea (mg/dL)	33 ± 8	36 ± 14	33 ± 14	33 ± 15	36 ± 11	38 ± 17
Creatinine (mg/dL)	0.92 ± 0.2	0.92 ± 0.2	0.96 ± 0.2	1.0 ± 0.33	0.95 ± 0.2	1.01 ± 0.3
Uric acid (mg/dL)	4.5 ± 1.6	4.0 ± 1.3	4.4 ± 1.8	3.8 ± 0.9	5.1 ± 1.9	4.6 ± 1.4
Total cholesterol (mg/dL)	200 ± 44	202 ± 69	196 ± 41	211 ± 70	198 ± 42	210 ± 47
Triglycerides (md/dL)	170 ± 69	147 ±46 *	219 ± 115	221 ± 122	197 ± 85	205 ± 62
HDL-cholesterol (mg/dL)	61 ± 26	56 ± 16	54 ± 16	53 ± 15	54 ± 17	52 ± 11
Albumin (g/dL)	4.3 ± 0.3	4.8 ± 0.6	4.4 ± 0.4	4.9 ± 0.5	4.3 ± 0.3	4.8 ± 0.5
CRP (mg/dL)	0.23 ± 0.3	0.22 ± 0.4	0.22 ± 0.2	0.33 ± 0.3	0.37 ± 0.4	0.48 ± 0.5 *
HbA1c (%)	7.9 ± 2.0	7.8 ± 1.8	8.0 ± 2.0	8.1 ± 1.5	7.7 ± 2.2	8.0 ± 1.7

HbA1c: glycosylated hemoglobin; CRP: C reactive protein. Data are presented as mean ± SD. ANOVA of repeated measures. Tukey test as post hoc, * *p* < 0.05.

**Table 3:** OS markers in the study groups: baseline and after six months.

Parameters	EG1000 (*n* = 37)		EG500 (*n* = 32)		Placebo (*n* = 28)	
	Baseline	Six months	Baseline	Six months	Baseline	Six months
LPO (µmol/L)	0.230 ± 0.08	0.212 ± 0.04	0.236 ± 0.09	0.229 ± 0.06	0.219 ± 0.07	0.282 ± 0.07 *
8-Iso (pg/mL)	60 ± 25	45 ± 27	56 ± 29	53 ± 38	61 ± 24	76 ± 35 †
GPx (UI/L)	7181 ± 3794	8362 ± 2910	8219 ± 3616	8926 ± 3010	7635 ± 2988	7329 ± 3049
Cat (×10^4^ UI/gHb)	3.0 ± 1	2.9 ± 1	2.9 ± 1	2.7 ± 1	2.9 ± 1	2.3 ± 1
TAC (µmol/L)	1003 ± 247	1225 ± 249 *	1016 ± 216	1014 ± 269	1004 ± 202	993 ± 217
SOD (Ul/L)	167 ± 15	173 ± 12	172 ± 16	178 ± 10	171 ± 15	167 ± 10 †
SOD/GPx ratio	0.030 ± 0.01	0.023 ± 0.01	0.027 ± 0.01	0.026 ± 0.01	0.026 ± 0.01	0.023 ± 0.01
GAP	283 ± 242	434 ± 234 ‡	288 ± 221	202 ± 284	276 ± 175	205 ± 229
OSS	2.5 ± 1	1.3 ± 1	2.0 ± 1	1.7 ± 1	1.9 ± 1	2.8 ± 1 *

LPO: lipoperoxides; 8-Iso: isoprostanes; GPx: glutathione peroxidase; Cat: catalase; TAC: total antioxidant capacity; SOD: superoxide dismutase; OSS: oxidative stress score. Data are presented as mean ± SD. ANOVA of repeated measures. Tukey test as post hoc, * *p* < 0.05 placebo vs. EG1000; † *p* < 0.05 placebo vs. (EG1000 and EG500); ‡ *p* < 0.05 EG1000 vs. (EG500 and placebo).

**Table 4:** Oxidative stress index in the study groups: baseline and after six months.

	EG1000 (*n* = 37)		EG500 (*n* = 32)		Placebo (*n* = 28)	
	Baseline	Six months	Baseline	Six months	Baseline	Six months
Without OS	5 (13%)	13 (35%) *	3 (10%)	9 (28%) *	6 (21%)	2 (7%) *
Mild OS	14 (38%)	18 (49%)	19 (59%)	15 (47%)	12 (43%)	8 (29%) *
Moderate OS	10 (27%)	5 (13%) *	9 (28%)	8 (25%)	8 (29%)	15 (54%) *
Severe OS	8 (22%)	1 (3%) *	1 (3%)	0 (0%)	2 (7%)	3 (10%)

OS: oxidative stress. McNemar test, * *p* < 0.05 baseline vs. after six months.

## Data Availability

The data presented in this study are available upon request from the corresponding authors.

## References

[1] American Diabetes Association (2018). Classification and diagnosis of diabetes: Standards of medical care in diabetes-2018. Diabetes Care.

[2] Sun H., Saeedi P., Karuranga S., Pinkepank M., Ogurtsova K., Duncan B.B., Stein C., Basit A., Chan J.C.N., Mbanya J.C. (2022). IDF Diabetes Atlas: Global, regional and country-level diabetes prevalence estimates for 2021 and projections for 2045. Diabetes Res. Clin. Pract..

[3] Deshpande A.D., Harris-Hayes M., Schootman M. (2008). Epidemiology of diabetes and diabetes-related complications. Phys. Ther..

[4] Yaribeygi H., Sathyapalan T., Atkin S.L., Sahebkar A. (2020). Molecular mechanisms linking oxidative stress and diabetes mellitus. Oxidative Med. Cell. Longev..

[5] Pang L., Lian X., Liu H., Zhang Y., Li Q., Cai Y., Ma H., Yu X. (2020). Understanding diabetic neuropathy: Focus on oxidative stress. Oxidative Med. Cell. Longev..

[6] Liguori I., Russo G., Curcio F., Bulli G., Aran L., Della-Morte D. (2018). Oxidative stress, aging, and diseases. Clin. Interv. Aging.

[7] Venkataraman K., Khurana S., Tai T.C. (2013). Oxidative stress in aging—Matters of the heart and mind. Int. J. Mol. Sci..

[8] Pannu N., Bhatnagar A. (2019). Resveratrol: From enhanced biosynthesis and bioavailability to multitargeting chronic diseases. Biomed. Pharmacother..

[9] Diaz-Gerevini G.T., Repossi G., Dain A., Tarres M.C., Das U.N., Eynard A.R. (2016). Beneficial action of resveratrol: How and why?. Nutrition.

[10] Koushki M., Amiri-Dashatan N., Ahmadi N., Abbaszadeh H.A., Rezaei-Tavirani M. (2018). Resveratrol: A miraculous natural compound for diseases treatment. Food Sci. Nutr..

[11] Meng X., Zhou J., Zhao C.N., Gan R.Y., Li H.B. (2020). Health benefits and molecular mechanisms of resveratrol: A narrative review. Foods.

[12] Galiniak S., Aebisher D., Bartusik-Aebisher D. (2019). Health benefits of resveratrol administration. Acta Biochim. Pol..

[13] Colica C., Milanović M., Milić N., Aiello V., De Lorenzo A., Abenavoli L. (2018). A Systematic review on natural antioxidant properties of resveratrol. Nat. Prod. Commun..

[14] Rauf A., Imran M., Suleria H.A.R., Ahmad B., Petersf D.G., Mubarak M.S. (2017). A comprehensive review of the health perspectives of resveratrol. Food Funct..

[15] Öztürka E., Karaboğa A.K.A., Yerer M.B., Bishayee A. (2017). Resveratrol and diabetes: A critical review of clinical studies. Biomed. Pharmacother..

[16] Watroba M., Szukiewicz D. (2016). The role of sirtuins in aging and age-related diseases. Adv. Med. Sci..

[17] Kitada M., Ogura Y., Monno I., Koya D. (2019). Sirtuins and type 2 diabetes: Role in inflammation, oxidative stress, and mitochondrial function. Front. Endocrinol..

[18] Goh K.P., Lee H.Y., Lau D.P., Supaat W., Chan Y.H., Yung A.F. (2014). Effects of resveratrol in patients with type 2 diabetes mellitus on skeletal muscle SIRT1 expression and energy expenditure. Int. J. Sport Nutr. Exerc. Metab..

[19] Samuel V.P., Gupta G., Dahiya R., Jain D.A., Mishra A., Dua K. (2019). Current update on preclinical and clinical studies of resveratrol, a naturally occurring phenolic compound. Crit. Rev. Eukaryot. Gene Expr..

[20] Park E.J., Pezzuto J.M. (2015). The pharmacology of resveratrol in animals and humans. Biochim. Biophys. Acta.

[21] Oyenihi O.R., Oyenihi A.B., Adeyanju A.A., Oguntibeju O.O. (2016). Antidiabetic effects of resveratrol: The way forward in its clinical utility. J. Diabetes Res..

[22] Singh A.P., Singh R., Singh S.V., Rai V., Kaschula C.H., Maiti P., Gupta S.C. (2019). Health benefits of resveratrol: Evidence from clinical studies. Med. Res. Rev..

[23] García-Martínez B.I., Ruiz-Ramos M., Pedraza-Chaverri J., Santiago-Osorio E., Mendoza-Núñez V.M. (2021). Hypoglycemic effect of resveratrol: A systematic review and meta-analysis. Antioxidants.

[24] Ahmed T., Javed S., Javed S., Tariq A., Šamec D., Tejada S., Nabavi S.F., Braidy N., Nabavi S.M. (2017). Resveratrol and Alzheimer’s disease: Mechanistic insights. Mol. Neurobiol..

[25] Berman A.Y., Motechin R.A., Wiesenfeld M.Y., Holz M.K. (2017). The therapeutic potential of resveratrol: A review of clinical trials. NPJ Precis. Oncol..

[26] Tonetto I.F.A., Baptista M.H.B., Gomides D.S., Pace A.E. (2019). Quality of life of people with diabetes mellitus. Rev. Esc. Enferm. USP.

[27] Dehdashtian E., Hossein M.P., Hemati K., Mehrzadi S., Hosseinzadeh A. (2020). Therapeutic application of nutraceuticals in diabetic nephropathy: Current evidence and future implications. Diabetes Metab. Res. Rev..

[28] Theodotou M., Fokianos K., Mouzouridou A., Konstantinou C., Aristotelous A., Prodromou D., Chrysokou A. (2017). The effect of resveratrol on hypertension: A clinical trial. Exp. Ther. Med..

[29] Cao X., Luo T., Tang Z. (2014). Resveratrol prevents Angll-induced hypertension via AMPK activation and RhoA/ROCK suppression in mice. Hypertens Res..

[30] Timmers S., Konings E., Bilet N., Houtkooeper R., Van Der Weijer T., Goossens G.H., Hoeks J., Van Der Kriekeen S., Ryu D., Kersten S. (2011). Calorie restriction-like effects of 30 days of resveratrol (resVida^TM^) supplementation on energy metabolism and metabolic profile in obese humans. Cell Metab..

[31] Méndez-del Villar M., González-Ortiz M., Martínez-Abundis E., Pérez-Rubio K., Lizárraga-Valdez R. (2014). Effect of resveratrol administration on metabolic syndrome, insulin sensitivity, and insulin secretion. Metab. Syndr. Relat. Disord..

[32] Gowd V., Kang Q., Wang Q., Wang Q., Chen F., Cheng K.W. (2020). Resveratrol: Evidence for its nephroprotective effect in diabetic nephropathy. Adv. Nutr..

[33] Mahjabeen W., Khan D.A., Mirza S.A. (2022). Role of resveratrol supplementation in regulation of glucose hemostasis, inflammation and oxidative stress in patients with diabetes mellitus type 2: A randomized, placebo-controlled trial. Complement. Ther. Med..

[34] Abdollahi S., Salehi-Abargouei A., Toupchian O., Sheikhha M.H., Fallahzadeh H., Rahmanian M., Tabatabaie M., Mozaffari-Khosravi H. (2019). The effect of resveratrol supplementation on cardio-metabolic risk factors in patients with type 2 diabetes: A randomized, double-blind controlled trial. Phytother. Res..

[35] Javid A.Z., Hormoznejad R., Yousefimanesh H., Zakerkish M., Haghighi-zadeh M.H., Dehghan P., Ravanbakhsh M. (2016). The impact of resveratrol supplementation on blood glucose, insulin, insulin resistance, triglyceride, and periodontal markers in type 2 diabetic patients with chronic periodontitis. Phytother. Res..

[36] Bhatt J.K., Thomas S., Nanjan M.J. (2012). Resveratrol supplementation improves glycemic control in type 2 diabetes mellitus. Nutr. Res..

[37] Hoseini A., Namazi G., Farrokhian A., Reiner Z., Aghadavod E., Bahmani F., Asemi Z. (2019). The effects of resveratrol on metabolic status in patients with type 2 diabetes mellitus and coronary heart disease. Food Funct..

[38] Khodabandehloo H., Seyyedebrahimi S., Esfahani E.N., Razi F., Meshkani R. (2018). Resveratrol supplementation decreases blood glucose without changing the circulating CD14+ CD16+ monocytes and inflammatory cytokines in patients with type 2 diabetes: A randomized, double-blind, placebo-controlled study. Nutr. Res..

[39] Hausenblas H.A., Schoulda J.A., Smoliga J.M. (2015). Resveratrol treatment as an adjunct to pharmacological management in type 2 diabetes mellitus—Systematic review and meta-analysis. Mol. Nutr. Food Res..

[40] Liu K., Zhou R., Wang B., Mi M.T. (2014). Effect of resveratrol on glucose control and insulin sensitivity: A meta-analysis of 11 randomized controlled trials. Am. J. Clin. Nutr..

[41] De Ligt M., Bruls Y., Hansen J., Habets M.F., Havekes B., Nascimento E.B.M., Moonen-Kornips E., Schaart G., Schrauwen-Hinderling V.B., Lichtenbelt W.V.M. (2018). Resveratrol improves ex vivo mitochondrial function but does not insulin sensitivity or brown adipose tissue in first degree relatives of patients with type 2 diabetes. Mol. Metab..

[42] Timmers S., De Ligt M., Phielix E., Van de Weijer T., Hansen J., Moonen-Kornips E., Schaart G., Kunz I., Hesselink M.K.C., Schrauwen-Hinderling V.B. (2016). Resveratrol as add-on therapy in subjects with well-controlled type 2 diabetes: A randomized controlled trial. Diabetes Care.

[43] Thazhath S.S., Wu T., Bound M.J., Checklin H.L., Standfield S., Jones K.L., Horowitz M., Rayner C.K. (2016). Administration of resveratrol for 5 wk has no effect on glucagon-like peptide 1 secretion, gastric emptying, or glycemic control in type 2 diabetes: A randomized controlled trial. Am. J. Clin. Nutr..

[44] Bo S., Ponzo V., Ciccone G., Evangelista A., Saba F., Goitre I., Procopio M., Pagano G.F., Cassader M., Gambino R. (2016). Six months of resveratrol supplementation has no measurable effect in type 2 diabetic patients. A randomized, double blind, placebo-controlled trial. Pharmacol. Res..

[45] Jeyaraman M.M., Al-Yousif N.S., Mann A.S., Dolinsky V.W., Rabbani R., Zarychanski R., Abou-Setta A.M. (2020). Resveratrol for adults with type 2 diabetes mellitus. Cochrane Database Syst. Rev..

[46] García-Martínez B.I., Ruiz-Ramos M., Pedraza-Chaverri J., Santiago-Osorio E., Mendoza-Núñez V.M. (2022). Influence of age and dose on the effect of resveratrol for glycemic control in type 2 diabetes mellitus: Systematic review and meta- analysis. Molecules.

[47] Zhang C., Luo J., Yu B., Chen J., Chen D. (2015). Effect of resveratrol on lipid metabolism in muscle and adipose tissues: A reevaluation in a pig model. J. Funct. Foods..

[48] Cao X., Liao W., Xia H., Wang S., Sun G. (2022). The effect of resveratrol on blood lipid profile: A dose-response Meta-analysis of randomized controlled trials. Nutrients.

[49] Lee C.C., Adler A.I., Sandhu M.S., Sharp S.J., Forouhi N.G., Erqou S., Luben R., Bingham S., Khaw K.T., Wareham N.J. (2009). Association of C-reactive protein with type 2 diabetes: Prospective analysis and meta-analysis. Diabetologia.

[50] Oguntibeju O.O. (2019). Type 2 diabetes mellitus, oxidative stress and inflammation: Examining the links. Int. J. Physiol. Pathophysiol. Pharmacol..

[51] Meng T., Xiao D., Muhammed A., Deng J., Chen L., He J. (2021). Anti-inflammatory action mechanisms of resveratrol. Molecules.

[52] Zhang L.-X., Li C.-X., Kakar M.U., Khan M.S., Wu P.-F., Amir R.M., Dai D.-F., Naveed M., Li Q.-Y., Saeed M. (2021). Resveratrol (RV): A pharmacology review and call for further research. Biomed. Pharmacother..

[53] Khattar S., Khan S.A., Zaidi S.A.A., Darvishikolour M., Farooq U., Naseef P.P., Kurunian M.S., Khan M.Z., Shamin A., Khan M.M.U. (2022). Resveratrol from dietary supplement to a drug candidate: An assessment of potential. Pharmaceuticals.

[54] Salehi B., Mishra A.P., Nigam M., Sener B., Kilic M., Sharifi-Rad M., Fokou P.V.T., Martins N., Sharifi-Rad J. (2018). Resveratrol: A double-edged sword in health benefits. Biomedicines.

[55] Sattarinezhad A., Roozbeh J., Yeganeh S., Omrani G., Shams M. (2019). Resveratrol reduces albuminuria in diabetic nephropathy: A randomized double-blind placebo-controlled clinical trial. Diabetes Metab..

[56] Collodel G., Moretti E., Noto D., Corsaro R., Signorini C. (2022). Oxidation of polyunsaturated fatty acids a promising area of research in infertility. Antioxidants.

[57] Sadi G., Konat D. (2016). Resveratrol regulates oxidative biomarkers and antioxidant enzymes in the brain of streptozotocin-induced diabetic rats. Pharm. Biol..

[58] Gu T., Wang N., Wu T., Ge Q., Chen L. (2021). Antioxidative stress mechanisms behind Resveratrol: A multidimensional analysis. J. Food Qual..

[59] Menshchikova E.B., Zenkov N.K., Tkachev V.O., Lemza A.E., Kandalintseva N.N. (2013). Protective effect of ARE-Inducing phenol antioxidant TS-13 in chronic inflammation. Bull. Exp. Biol. Med..

[60] Kou X., Kirberger M., Yang Y., Chen N. (2013). Natural products for cancer prevention associated with Nrf2-ARE pathway. Food Sci. Hum. Wellness.

[61] Seyyedebrahimi S., Khodabandehloo H., Esfahani E.N., Meshkani R. (2018). The effects of resveratrol on markers of oxidative stress in patients with type 2 diabetes: A randomized, double-blind, placebo-controlled clinical trial. Acta Diabetol..

[62] Sánchez-Rodríguez M.A., Santiago-Osorio E., Vargas L.A., Mendoza-Núñez V.M. (2004). Propuesta de un constructo para evaluar integralmente el estrés oxidativo. Bioquimia.

[63] DiNicolantonio J.J., McCarty M.F., O’Keefe J.H. (2022). Nutraceutical activation of Sirt1: A review. Open Heart.

[64] Grzeczka A., Kordowitzki P. (2022). Resveratrol and SIRT1: Antiaging cornerstones for oocytes?. Nutrients.

[65] Song J., Yang B., Jia X., Li M., Tan W., Ma S., Shi X., Feng L. (2018). Distinctive roles of sirtuins on diabetes, protective or detrimental?. Front. Endocrinol..

[66] Houtkooper R.H., Pirinen E., Auwerx J. (2016). Sirtuins as regulators of metabolism and healthspan. Nat. Rev. Mol. Cell Biol..

[67] Xu J., Kitada M., Koya D. (2020). The impact of mitochondrial quality control by sirtuins on the treatment of type 2 diabetes and diabetic kidney disease. Biochim. Biophys. Acta Mol. Basis Dis..

[68] DiNicolantonio J.J., McCarty M.F., Assanga S.I., Lujan L.L., O’Keefe J.H. (2022). Ferulic acid and berberine, via SIRT1 and AMPK, may act as cell cleasing promoters of healthy longevity. Open Heart.

[69] Khan M.A., Chen H., Wan X., Tania M., Xu A., Chen F., Zhang D. (2013). Regulatori effects of resveratrol on antioxidant enzymes: A mechanism of growth inhibition and apoptosis induction in cancer cells. Mol. Cells..

[70] Olmos Y., Sánchez-Gómez F.J., Wild B., García-Quintans N., Cabezudo S., Lamas S., Monsalve M. (2013). SirT1 regulation of antioxidant genes is dependent on the formation of a FOXO3a/PGC-1α complex. Antioxid. Redox Signal..

[71] Hwang J., Yao H., Caito S., Sundar I.K., Rahman I. (2013). Redox regulation of SIRT1 in inflammation and cellular senescence. Free. Radic. Biol. Med..

[72] Chachay V.S., Kirkpatrick C.M., Hickman I.J., Ferguson M., Prins J.B., Martin J.H. (2011). Resveratrol–pills to replace a healthy diet?. Br. J. Clin. Pharmacol..

[73] Ramírez-Garza S.L., Laveriano-Santos E.P., Marhuenda-Muñoz M., Storniolo C.E., Tresserra-Rimbau A., Vallverdú-Queralt A., Lamuela-Raventós R.M. (2018). Health effects of resveratrol: Results from human intervention trials. Nutrients.

[74] de Salud S. (1999). Norma Oficial Mexicana NOM-030-SSA2-1999, Para la Prevención, Tratamiento y Control de la Hipertensión Arterial.

[75] Jentzsch A.M., Bachmann H., Fürst P., Biesalski H.K. (1996). Improved analysis of malondialdehyde in human body fluids. Free. Radic. Biol. Med..

[76] McCord J.M., Fridovich I. (1969). Superoxide dismutase an enzyme function for erythrocuprein. J. Biol. Chem..

[77] Plagia D.E., Valentine W.N. (1967). Studies on the quantitative characterization of erytrocyte of glutathione peroxidase. J. Lab. Clin. Med..

[78] Aebi H. (1984). Catalase in vitro. Methods Enzymol..

[79] Miller N.J., Rice E.C., Davies M.J. (1994). Total antioxidant status in plasma and body fluids. Methods Enzymol..

